# The Possible Roles of IL-4/IL-13 in the Development of Eosinophil-Predominant Severe Asthma

**DOI:** 10.3390/biom14050546

**Published:** 2024-05-02

**Authors:** Kazuyuki Nakagome, Makoto Nagata

**Affiliations:** 1 Department of Respiratory Medicine, Saitama Medical University, Saitama 350-0495, Japan; 2 Allergy Center, Saitama Medical University, Saitama 350-0495, Japan

**Keywords:** bronchial asthma, IL-4, IL-13

## Abstract

Bronchial asthma is characterized by airway inflammation, airway hyperresponsiveness, and reversible airway obstruction. Eosinophils contribute to the pathogenesis of airway disease mainly by releasing eosinophil-specific granules, lipid mediators, superoxide anions, and their DNA. Type-2 cytokines such as interleukin (IL)-4 and IL-13 also play roles in the development of bronchial asthma. Among these cytokines, IL-4 is involved in T-cell differentiation, B-cell activation, B-cell differentiation into plasma cells, and the production of immunoglobulin E. Although IL-13 has similar effects to IL-4, IL-13 mainly affects structural cells, such as epithelial cells, smooth muscle cells, and fibroblasts. IL-13 induces the differentiation of goblet cells that produce mucus and induces the airway remodeling, including smooth muscle hypertrophy. IL-4 and IL-13 do not directly activate the effector functions of eosinophils; however, they can induce eosinophilic airway inflammation by upregulating the expression of vascular cell adhesion molecule-1 (for adhesion) and CC chemokine receptor 3 ligands (for migration). Dupilumab, a human anti-IL-4 receptor α monoclonal antibody that inhibits IL-4 and IL-13 signaling, decreases asthma exacerbations and mucus plugs and increases lung function in moderate to severe asthma. In addition, dupilumab is effective for chronic rhinosinusitis with nasal polyps and for atopic dermatitis, and IL-4/IL-13 blocking is expected to suppress allergen sensitization, including transcutaneous sensitization and atopic march.

## 1. Introduction

Bronchial asthma is characterized by eosinophilic airway inflammation, airway hyperresponsiveness (AHR), and reversible airway obstruction [1]. Various types of cells contribute to the pathophysiology of airway inflammation in asthma. Among these, eosinophils play important roles in the pathogenesis of asthma, primarily through the release of eosinophil-specific granules, including major basic protein (MBP), lipid mediators, and their DNA, and the generation of superoxide anions (O_2_^−^) [2].

Type-2 cytokines, such as interleukin (IL)-4, IL-5, and IL-13, also contribute to the development of bronchial asthma [3,4]. For example, IL-5 plays a crucial role in the differentiation of eosinophils in bone marrow, their activation, and their survival at the site of inflammation, including the airways. IL-4 is involved in T-cell differentiation, B-cell activation, B-cell differentiation into plasma cells, and the production of immunoglobulin (Ig) E, which contributes to allergic responses by enhancing the crosslinking of allergens on mast cells or basophils [5]. Although IL-13 has similar effects to IL-4, the clinically meaningful effects of IL-13 are found on structural cells, such as epithelial cells, smooth muscle cells, and fibroblasts [5]. For example, IL-13 induces the differentiation of goblet cells that produce mucus and are involved in remodeling, including smooth muscle hypertrophy [5], which is discussed below. IL-13 is not involved in T-cell differentiation, as naïve T-cells do not express IL-13 receptors [6]. Some bacterial lysates and herbal medicines can suppress IL-4/IL-13 expression and eosinophilic airway inflammation [7,8].

There are two types of receptors (Rs) for IL-4/IL-13: type-I IL-4R consists of IL-4Rα and a common gamma chain (γc), while type-II IL-4R consists of IL-4Rα and IL-13Rα1. IL-4 can bind to type-I and type-II IL-4Rs, while IL-13 binding is limited to type-II IL-4R. IL-4Rα/γc is highly expressed mainly on hematopoietic cells; however, IL-4Rα/IL-13Rα1 is highly expressed on cells other than hematopoietic cells, which may account for the effects of IL-13 on non-hematopoietic cells, including airway epithelial cells, endothelial cells, and smooth muscle cells.

In the lungs, type-2 cytokines, including IL-4, IL-5, and IL-13, are mainly produced by T helper (Th) 2 cells and T follicular helper cells [9]. Basophils and mast cells also produce IL-4, IL-5, and IL-13, and eosinophils produce IL-13. However, recent studies have suggested that cells other than Th2 cells produce type-2 cytokines. For example, Th1 cells treated with IL-18 and antigens produce not only type-1 cytokines, including interferon (IFN)-γ, but also type-2 cytokines, including IL-13 [10]. Th1 cells with chronic antigen stimulation produce IL-13 through the expression of E4BP4 [11]. Furthermore, group-2 innate lymphoid cells (ILC2s) produce large amounts of IL-5 and IL-13 in response to IL-33, IL-25, and thymic stromal lymphopoietin (TSLP), as described below [4,12]. ILC2s are thought to be a major source of type-2 cytokines in the airways of patients with severe asthma [13].

In this review, we discuss the possible roles of IL-4/IL-13 in the development of eosinophil-predominant severe asthma.

## 2. Role of Eosinophils in the Pathogenesis of Asthma

In severe asthma cases, eosinophils remain in the airways even during treatment with inhaled corticosteroids (ICS) [14], suggesting the contribution of eosinophils to the pathogenesis of severe asthma. Regarding the role of eosinophils in the disease activity of asthma, the sputum eosinophil ratio and the blood eosinophil count are already known to be biomarkers for predicting asthma exacerbation [15,16,17]. Asthma exacerbation can be suppressed by controlling the sputum eosinophil ratio [15]. Furthermore, the blood eosinophil count is associated with the frequency of asthma exacerbations [16]. We retrospectively investigated the association between asthma exacerbations and the blood eosinophil count or fractional exhaled nitric oxide (FeNO) in severe asthma cases and found that severe exacerbations are more common in asthma cases with a high blood eosinophil count (>300 cells/μL) [17].

As for the mechanisms of increased asthma exacerbation in eosinophil-predominant asthma, there was a correlation between the AHR and MBP concentration and the ratio of eosinophils and airway epithelial cells in bronchoalveolar lavage fluid (BALF) [18], suggesting that MBP-induced injury of the airway epithelial cells may be involved in the development of AHR and thus the exacerbation of asthma. Recently, the role of eosinophils in hypersensitivity to external stimuli through airway sensory innervation has been highlighted. For example, airway sensory nerve density correlates with blood eosinophil count and the expression of eosinophil-derived neurotoxin (EDN) in the airway in asthmatics [19]. Furthermore, airway nerve density is also increased in IL-5 transgenic mice [19]. Therefore, eosinophils can contribute to the pathogenesis of asthma exacerbation according to various mechanisms, including increased AHR and hypersensitivity to external stimuli.

Moreover, eosinophils play important roles in the development of airway remodeling in asthma [20,21,22]. For example, Humbles et al. reported that mice with selective ablation of the eosinophil lineage do not develop collagen deposition or increases in the airway smooth muscle cells in response to allergens, indicating the critical role of eosinophils in the development of airway remodeling [20]. Human eosinophils can induce the epithelial–mesenchymal transition through transforming growth factor (TGF)-β1 [21]. We reported that in vivo IL-5 gene delivery induces eosinophilia and enhances the expression of TGF-β, whose signal is transduced to other cells [22]. These findings suggested that eosinophils can induce airway remodeling through TGF-β.

## 3. Mechanism of Eosinophilic Airway Inflammation including the Role of IL-4/IL-13

IL-5 is the most important cytokine for eosinophil differentiation and activation in asthma. IL-5 induces eosinophil differentiation mainly in the bone marrow and activates eosinophils and promotes their survival at the site of inflammation, including the airways, after their migration across the endothelial cells. Indeed, anti-IL-5 treatment, such as with an anti-IL-5 antibody (Ab) or an anti-IL-5R Ab, reduces the blood eosinophil count and asthma exacerbation frequency in patients with severe asthma with an increased blood eosinophil count [23].

However, IL-5 alone cannot be the cause. For example, IL-5 does not directly induce eosinophil migration into the airways. Furthermore, an anti-IL-5 Ab may be insufficient to suppress eosinophil infiltration and activation in the airways. Kelly et al. have reported that anti-IL-5 monoclonal (m) Ab treatment did not inhibit the release of eosinophil specific-granule proteins and did not completely suppress eosinophil accumulation in the airways after allergen challenge [24]. We have reported that among severe asthma cases with high FeNO (>27 ppb) and a low blood eosinophil count (<265 cells/μL), with the treatment including an anti-IL-5/IL-5R Ab, almost half of the cases demonstrated sputum eosinophilia (>2.7%) [25]. Therefore, mechanisms other than those involving IL-5 also contribute to the pathogenesis of eosinophilic airway inflammation. For example, CC chemokine receptor 3 (CCR3) ligands, mainly induced by IL-4/IL-13, are involved in the accumulation of eosinophils in the airways. Furthermore, granulocyte–macrophage colony-stimulating factor (GM-CSF) or IL-3 may have the same effect as IL-5 on eosinophil activation and cell survival, as discussed below.

**Figure 1:**
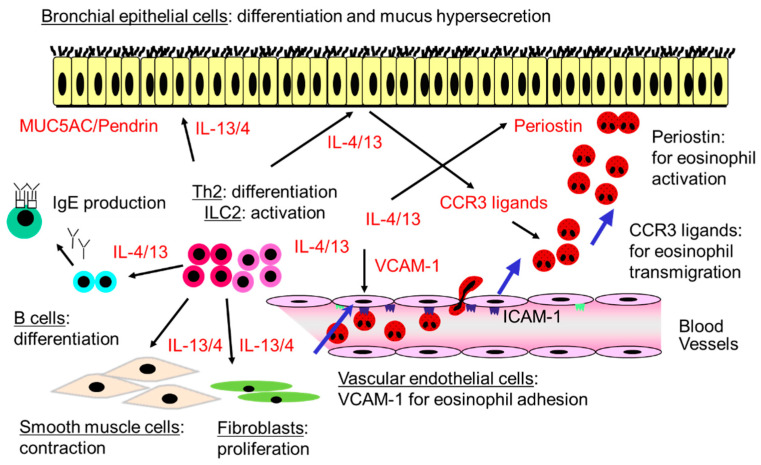
Possible roles of IL-4/IL-13 in the development of eosinophil-predominant severe asthma. IL-4 plays an important role in the differentiation of Th2 and the activation of ILC2s and thus in allergic sensitization and maintenance. IL-13/IL-4 induces the differentiation of bronchial epithelial cells and mucus production through the upregulation of MUC5AC and pendrin and also enhances the contractility of airway smooth muscle cells and induces the proliferation of fibroblasts. IL-4/IL-13 plays a role in the development of eosinophilic airway inflammation by increasing the expression of VCAM-1 in vascular endothelial cells; the expression of CCR3 ligands, which contribute to the migration of eosinophils into the airways; and the expression of periostin, which can activate eosinophils in the airways. These mechanisms are involved in IL-4/IL-13-mediated eosinophil-predominant severe asthma.

Not only IL-4/IL-13 but also IL-4/IL-13-induced chemokines play roles in the adhesion of eosinophils to vascular endothelial cells and the accumulation of eosinophils in the airways. Eosinophils first need to attach to vascular endothelial cells to migrate across them [26,27]. Vascular cell adhesion molecule (VCAM)-1 on vascular endothelial cells plays an important role in selective eosinophil recruitment. IL-4/IL-13 induces the expression of VCAM-1 on vascular endothelial cells (Figure 1), which blood eosinophils adhere to spontaneously. Fukuda et al. have reported that the expression of VCAM-1 on vascular endothelial cells is correlated with the IL-4 concentration in BALF or the eosinophil count in airway tissues [28]. Furthermore, VCAM-1 directly activates the functions of eosinophils, including the generation of O_2_^−^ and the release of specific granules, in what may be the first activation step during the process of eosinophil accumulation in the airways [26,27].

After the adhesion of eosinophils to VCAM-1-expressing endothelial cells, CC chemokines, particularly CCR3 ligands, induce eosinophil migration into the airways (Figure 1) [26,27]. CCR3 ligands, including eotaxin; eotaxin-2; regulated upon activation, normal T-expressed and secreted (RANTES); and monocyte chemotactic protein (MCP)-3 and MCP-4, which can be upregulated by IL-4/IL-13, effectively promote the transmigration of eosinophils across VCAM-1-expressing endothelial cells [29]. It has been reported that the levels of CCR3 ligands, including eotaxin, are increased in the airways of asthma or eosinophilic pneumonia patients [30] and that anti-CCR3 Abs, but not anti-IL-5 Abs, inhibit the eosinophil transendothelial migration induced by the BALF from subjects with acute eosinophilic pneumonia [30]. These findings suggest that the CCR3 ligands induced by IL-4/IL-13, rather than IL-5, contribute to eosinophil migration in the airways.

Periostin, an extracellular matrix protein, plays a role in airway remodeling in eosinophil-predominant asthma and is known to be a biomarker of eosinophilic inflammation or type-2-mediated immune responses and airway remodeling in bronchial asthma [31]. Indeed, the expression of periostin is increased in the airways of asthma patients and can be induced by IL-13/IL-4 in vitro [31]. Periostin also acts as a matricellular protein that can directly activate various types of cells [31]. For example, we have reported that periostin can activate eosinophils and induce adhesion, the generation of O_2_^−^, and specific granule protein release through the αMβ2 integrin (Figure 1) [32]. Consequently, the enhanced expression of periostin induced by IL-13/IL-4 may contribute to eosinophil activation.

In summary, in the pathogenesis of eosinophilic airway inflammation, IL-4/IL-13 first induces the expression of VCAM-1 in the vascular endothelial cells and then increases the expression of the CCR3 ligands that contribute to the migration of eosinophils into the airways (Figure 1). Furthermore, IL-4/IL-13 induces the expression of periostin, which can activate eosinophils (Figure 1). Therefore, IL-4/IL-13 blocking may decrease the airway eosinophil count; however, it may also increase the blood eosinophil count by suppressing the expression of VCAM-1 in the endothelial cells and the migration of eosinophils into the airways.

Regarding mechanisms other than those involving IL-4/IL-13, GM-CSF and/or IL-3 contribute to the activation of eosinophils after the migration process, even in the absence of IL-5. GM-CSF can directly activate eosinophil effector functions, including O_2_^−^ generation and specific granule protein release, in vitro [33]. The eosinophils in the airways of asthmatic patients after partial allergen challenge are activated by GM-CSF but not by IL-5, probably due to the downregulation of IL-5Rα in airway eosinophils [34]. Additionally, lipid mediators, including cysteinyl leukotrienes (cysLTs), are involved in the infiltration of eosinophils into the airways. The inhalation of leukotriene E4 (LTE4) enhances the accumulation of eosinophils. On the other hand, leukotriene D4 (LTD4) increases the expression of the β2 integrin on eosinophils and thereby enhances eosinophil adhesion [35]. Furthermore, LTD4 promotes the transendothelial migration of eosinophils, O_2_^−^ generation, and the release of specific granule proteins, mainly through cysLT1 receptors and β2 integrins [36].

Innate immune responses are also involved in the pathogenesis of eosinophilic airway inflammation; this process includes ILC2s as well as epithelial-cell-related cytokines such as IL-33, TSLP, and IL-25 [4,12]. The ILC2s activated by IL-33, TSLP, and IL-25 can produce IL-5 and IL-13 and thus induce eosinophilic inflammation. Several reports suggest that ILC2s are increased in the blood or airways of patients with asthma [37,38] and highly increased in severe asthma as compared with mild asthma [13,39]. ILC2s in severe asthma are more activated than in mild asthma [13,38]. For example, IL-5^+^ ILC2s in the peripheral blood and sputum of severe asthma patients are increased as compared to those with mild asthma or control patients [13]. Furthermore, IL-13^+^ ILC2s are increased in the peripheral blood of patients with uncontrolled asthma. TSLP contributes to the pathogenesis of corticosteroid-resistant airway inflammation according to Bcl-xL expression via the ILC2s [40,41]. Therefore, ILC2s contribute to the development of airway inflammation in severe asthma.

## 4. Attempts at Preventing and Targeting Eosinophilic Airway Infiltration in Severe Asthma

Considering the importance of eosinophilic airway inflammation in asthma, treatment strategies targeting eosinophils are evolving. Representative is the direct suppression of eosinophilic inflammation via anti-IL-5 treatment. As described above, in patients with severe asthma and an increased eosinophil count in the sputum or blood with frequent exacerbations, an anti-IL-5 Ab or an anti-IL-5R Ab reduces the blood eosinophil count and asthma exacerbation frequency [23]. An anti-IL-5R Ab attenuated mannitol-induced AHR in severe uncontrolled eosinophilic asthma [42]. Anti-IL-5 therapy also improves capsaicin cough sensitivity in patients with severe asthma [43]. Furthermore, an anti-IL-5 mAb significantly reduced the number of eosinophils expressing mRNA for TGF-β, the concentration of TGF-β protein in BALF, and the deposition of extracellular matrix proteins in bronchial tissue [44]. Furthermore, an anti-IL-5R Ab reduced the airway smooth muscle mass and myofibroblast count in the lamina propria in asthmatic airways [45], suggesting anti-IL-5 treatment can improve airway remodeling. Based upon these findings, we can now prescribe anti-IL-5 Abs/anti-IL-5R Abs to patients with eosinophil-predominant severe asthma.

The indirect suppression of eosinophilic inflammation using anti-IL-4/anti-IL-13 treatment is a promising treatment strategy, which is discussed below. Anti-IL-4/IL-13 treatment reduces airway eosinophilia despite a modest increase in the blood eosinophil count [46]. The suppression of eosinophilic inflammation using anti-TSLP treatment is also another promising treatment. It suppresses eosinophilic airway inflammation [47] and asthma exacerbation [48] in patients with severe asthma. Further research is needed to determine whether anti-TSLP treatment is more effective than anti-IL-5 treatment or not. We can now prescribe anti-IL-4R Abs or anti-TSLP Abs to patients with severe asthma.

## 5. Direct Effects of IL-13 on the Airway Structural Cells

The effects of IL-13 on the airway structural cells, such as airway epithelial cells, smooth muscle cells, and fibroblasts, have been established. IL-13 induces the differentiation of the bronchial epithelial cells and mucus production in vitro. For example, IL-13 increases the secretion of the cysteine-rich MUC5AC mucin from the airway epithelial cells (Figure 1) and the transportation of thiocyanate to the airways [49]. Furthermore, IL-13 induces pendrin, a molecule responsible for airway mucus production (Figure 1) [50]. Pendrin, which is induced on the apical side of the airway epithelial cells, increases mucus production and the expression of MUC5AC. IL-13 also induces the expression of transcription factor FOXA3 in the airway epithelial cells to regulate goblet cell metaplasia [51]. In addition, IL-13 induces the expression of SAM pointed domain-containing ETS transcription factor (SPDEF), which is associated with the expression of MUC5AC [52]. Moreover, the intratracheal administration of IL-13 increases AHR and induces mucus hypersecretion in mice in a manner that is independent of eosinophils and IgE [53].

Furthermore, IL-13 and IL-4 can induce the contraction of the smooth muscle cells as well as the proliferation of fibroblasts. Risse et al. have reported that IL-13 enhances the contractility of human airway smooth muscle cells (Figure 1) [54]. Manson et al. have reported that IL-4 or IL-13 augments histamine-, carbachol-, and LTD4-induced contraction of human airway smooth muscle cells [55]. Furthermore, IL-4 and IL-13 increase fibroblast growth factor 2-induced bronchial smooth muscle cell proliferation [56]. As for fibroblasts, Kraft et al. reported that IL-4 and IL-13 increase the proliferation of airway fibroblasts in patients with mild asthma [57]. IL-4 induces the proliferation of conjunctival fibroblasts and also increases the release of procollagen type I C-peptide and fibronectin and the deposition of collagen type III by fibroblasts [58]. IL-4 induces eotaxin-1 expression in fibroblasts [59]. Periostin is a component of the subepithelial fibrosis of bronchial asthma, as described above. IL-4 and IL-13 induce periostin in the airway epithelial cells and fibroblasts and thus play roles in the development of airway remodeling [60]. Moreover, Shoda et al. reported that IL-4 and IL-13 induce periostin production not only in primary normal human fibroblasts but also in microvascular endothelial cells derived from the lungs [61].

Therefore, IL-13 directly works on epithelial cells, smooth muscle cells, and fibroblasts (Figure 1) and is involved in the development of mucus hypersecretion, AHR, and airway remodeling.

## 6. The Role of IL-4 in Th2 Differentiation, ILC2 Activation, and the Maintenance of Allergic Responses

IL-4 plays a central role in the development of allergen sensitization and the differentiation of Th2 cells (Figure 1). Dendritic cells (DCs) present antigens to naïve T cells and then induce Th2 differentiation with IL-4. In a mouse model, the depletion of DCs in the effector phase suppressed allergic airway inflammation [62], suggesting that DCs, and thus allergen sensitization, play crucial roles in not only the induction but also the maintenance of allergic airway inflammation. Previously, we have reported that systemic allergen sensitization alone induces AHR prior to the establishment of eosinophilic airway inflammation in a mouse model and that IL-4 plays an important role in this process [63]. In humans, AHR is sometimes seen in cases of atopic dermatitis without asthma, suggesting that allergen sensitization may contribute to AHR. Furthermore, AHR due to allergen sensitization alone can be induced by the transfer of sensitized splenocytes, and allergen-sensitized IL-4/IL-13-producing CD4^+^CD62L^low^ memory/effector Th2 cells play an essential role in the development of transfer-induced AHR [64]. Notably, this type of AHR is not induced by the transfer of sensitized splenocytes from IL-4-deficient mice or from anti-IL-4-Ab-treated mice [64], suggesting that IL-4 plays an important role. As mentioned above, Th2 cells are activated during the process of allergen sensitization, and the IL-4 released from the Th2 cells further contributes to allergen sensitization and the maintenance of allergic responses.

IL-4 and IL-13 induce an alternative activation program in macrophages and play roles in their polarization to M2 macrophages. M2 macrophages are thought to contribute to the modulation of airway inflammation and tissue repair; however, their role in the development of severe asthma has not been fully clarified. IL-4-stimulated dermal-tissue-resident macrophages, in cooperation with IL-10, produce a large amount of eotaxin-2 [65], which can be functioned to amplify eosinophil influx.

IL-4 also plays a role in the development of innate immune responses and ILC2 activation (Figure 1). For example, in a system with the intratracheal administration of papain, IL-4 from the basophils activate ILC2s and induce eosinophilic airway inflammation [66]. Basophils are an important source of IL-4, and lung ILC2s express IL-4R [66]. Indeed, IL-4, in cooperation with IL-33, is found to directly activate lung ILC2s in vitro [66].

## 7. Effects of IL-4/IL-13 Blocking on Severe Asthma

Dupilumab is a human mAb for IL-4Rα that blocks the signals of IL-4 and IL-13. It is effective for severe asthma and is prescribed in the clinical setting. In the QUEST phase 3 randomized, double-blind, placebo-controlled clinical trial, dupilumab reduced asthma exacerbations and increased the lung function in moderate to severe asthma cases that were uncontrolled with moderate to high-dose ICS plus other controllers [67]. Furthermore, it also reduces the dose of oral corticosteroids. Dupilumab was particularly effective in patients with an elevated peripheral blood eosinophil count or with elevated FeNO. Dupilumab also significantly decreases FeNO and serum IgE, periostin, thymus and activation-regulated chemokine (TARC), and eotaxin-3 concentrations [67], confirming that these biomarkers are regulated by IL-4/IL-13. Rabe et al. recently reported that the effects of dupilumab in suppressing exacerbations and improving lung function increase as the peripheral blood eosinophil count increases [68]. Some bacterial lysates and herbal medicines can suppress IL-4/IL-13 expression and eosinophilic airway inflammation [7,8].

In several case reports, it was found that dupilumab reduces mucus plugs [69,70]. Mucus plugs are an important treatment target, as they are associated with asthma exacerbations and airflow limitation [49,71]. For example, Svenningsen et al. assessed ventilation heterogeneity using ^3^He magnetic resonance imaging as an index of mucus plugs and reported a case in which dupilumab improved ventilation heterogeneity that was unresponsive to anti-IgE Ab or anti-IL-5 Ab treatment [69]. A recent study has reported that dupilumab suppresses mucus plugs and ventilation defects in patients with moderate to severe asthma [72]. Furthermore, Tajiri et al. reported that dupilumab suppresses radiological mucus scores, as well as airway wall thickening [73]. In addition, IL-13 increases the secretion of the cysteine-rich MUC5AC mucin from the airway epithelial cells and the transportation of thiocyanate to the airways, as was described above [49]. Furthermore, IL-13 induces pendrin, which increases mucus production and MUC5AC expression [50]. Therefore, blocking of the IL-13 signal using dupilumab suppresses the development of mucus plugs, which is associated with the suppression of exacerbations and an improvement of airflow limitation.

The effect of dupilumab on maintaining improved lung function has also been reported. As a result of previous studies, such as QUEST (1 year; 52 weeks), dupilumab has been used for about 2 years (96 weeks) in patients that have a blood eosinophil count of more than 150 cells/μL or FeNO of more than 25 ppb, and its therapeutic effects were investigated in the TRAVERSE study. Dupilumab significantly improves the forced expiratory volume (approximately 0.35 L), and this effect persists for about 3 years [74]. The blood eosinophil counts were transiently elevated, but they eventually decreased to levels comparable to baseline levels. A recent study has reported that dupilumab reduces airway eosinophilia despite a modest increase in the blood eosinophil count [46]. Eosinophils have been shown to play important roles in airway remodeling [20,21,22]. Furthermore, dupilumab inhibits the effects of IL-13 and IL-4 on human airway smooth muscle cells in vitro [55]. Therefore, dupilumab may improve lung function though various mechanisms, such as by controlling smooth muscle cell contraction, mucus plugs, and airway eosinophilia.

The presence of eosinophilic chronic rhinosinusitis (ECRS) or nonsteroidal anti-inflammatory drug-exacerbated respiratory disease (NERD), a disease that often complicates ECRS, is strongly associated with asthma exacerbation or the worsening of asthma control [75,76]. Sinus surgery for ECRS reduces the urinary LTE4 levels in asthma patients with ECRS or NERD [77]. Therefore, controlling ECRS is an important strategy for controlling severe asthma complicated by ECRS. The serum periostin concentration is a biomarker for ECRS with asthma, and it is correlated with the Lund–Mackay score [78]. Cases with asthma complicated by ECRS have higher serum periostin concentrations than cases with asthma without ECRS, suggesting the involvement of IL-4/IL-13 in the pathogenesis of ECRS in asthma cases. Dupilumab reduces the polyp size, sinus opacification, and severity of symptoms in adult patients with severe ECRS with nasal polyps [79] and is prescribed for ECRS in the clinical setting. Also, dupilumab suppresses the annual severe asthma exacerbation rate more in asthma patients with ECRS than in those without ECRS [80]. Therefore, dupilumab plays an important role in the treatment of asthma and ECRS, as it is effective for not only asthma but also for ECRS. As for NERD, dupilumab suppresses urinary LTE4 production and improved asthma symptoms in patients with NERD [81]. Furthermore, recent studies have suggested that dupilumab increases aspirin tolerance in patients with NERD [82,83], suggesting that IL-4/IL-13 contributes to the pathogenesis of NERD.

Lebrikizumab is a humanized mAb that binds soluble IL-13 at the non-receptor-binding domain with a high affinity [84]. Bound IL-13 is able to form a complex with IL-13Rα1, whereas it prevents heterodimerization with IL-4Rα and prevents signal transduction. Therefore, lebrikizumab inhibits the IL-4Rα–IL-13Rα1 signaling complex while continuing to regulate endogenous IL-13 via the stimulation of IL-13Rα2 [84]. Lebrikizumab is effective against atopic dermatitis and can be used clinically. Corren et al. examined the effect of lebrikizumab on asthma that was inadequately controlled despite ICS in a phase 2 study [85]. They found that lebrikizumab increased FEV1 in severe asthma. Furthermore, patients with higher serum levels of periostin before treatment had a greater improvement in lung function with lebrikizumab than patients with low periostin levels (8.2 vs. 1.6% higher than in the placebo group), and there was a greater reduction in FeNO levels in the high-periostin subgroup [85]. Furthermore, Hanania et al. reported that lebrikizumab decreased the rate of asthma exacerbations, which was more pronounced in the periostin-high patients (60% reduction) than in the periostin-low patients (5% reduction) [86]. Lebrikizumab also increased lung function, with the greatest increase in FEV1 in the periostin-high patients (9.1% improvement) compared with the periostin-low patients (2.6% improvement) [86]. The effect of lebrikizumab on asthma exacerbation in biomarker-high patients with severe asthma (periostin ≥50 ng/mL or blood eosinophils ≥300 cells/μL) was later examined in phase 3 trials, LAVOLTA I and LAVOLTA II [87]. Although lebrikizumab significantly reduced asthma exacerbation in LAVOLTA I, its reduction was not slightly significant in LAVOLTA II (*p* = 0.06) [87]. As a result of these results, the development of a drug for asthma was terminated. However, Corren et al. recently carried out a post hoc analysis of three phase 3 clinical trials of lebrikizumab in adults and adolescents with uncontrolled asthma [88]. They found that lebrikizumab significantly reduced asthma exacerbations in a subpopulation of patients with elevated blood eosinophils (>300 cells/μL), elevated FeNO (>50 ppb), and a history of one or more asthma exacerbation in the preceding year [88], suggesting that the failure to demonstrate the efficacy of lebrikizumab described above was related to the patient selection in those trials.

Tralokinumab is a humanized mAb that competitively blocks the binding of IL-13 to both the IL-13Rα1 and IL-13Rα2 receptor chains [84]. Tralokinumab is also effective against atopic dermatitis and can be used clinically. Brightling et al. demonstrated in a phase 2b study that tralokinumab is tolerable and safe and slightly reduced asthma exacerbation [89]. Some partially encouraging results were found in a subgroup of patients with a higher baseline amount of dipeptidyl peptidase-4 and periostin, suggesting a possible treatment effect in certain populations. In a phase 3 study including STRATOS 1 and STRATOS 2, tralokinumab reduced the asthma exacerbation in patients with severe asthma, with baseline FeNO of 37 ppb or higher in STRATOS 1 but not in STRATOS 2 [90]. As a result of these results, the development of a drug for asthma was terminated.

Anrukinzumab is a humanized anti-IL-13 mAb which acts to block the cytokine and prevent the activation of IL-13Rα1 and IL-13Rα2. Anrukinzumab has been tested in asthma and ulcerative colitis in phase II studies [91]. However, no further research seems to be underway.

## 8. The Effects of Switching to Dupilumab in Asthma Patients Who Have an Inadequate Response to Anti-IL-5 Treatment

There have been several studies that have reported on the effects of switching to dupilumab in asthmatics who have had an insufficient response to anti-IL-5 treatment. For example, Bavaro et al. switched the treatment to dupilumab in 27 patients with NERD among 41 patients with an inadequate response to anti-IL-5/IL-5R Abs; the treatment (anti-IL-5/IL-5R Ab) was not changed for the remaining 14 patients since they did not consent to the change. Comparisons of the two groups revealed that switching to dupilumab improved lung function, asthma exacerbations, and sinusitis in the patients [92]. Similarly, Mümmler et al. switched the treatment to dupilumab in 38 asthma patients who had an inadequate response to other biologics (32 of these patients had an inadequate response to anti-IL-5 Abs), and they found that switching to dupilumab improved the lung function and asthma exacerbations in the patients [93]. Furthermore, FeNO of more than 25 ppb is found to predict a good response to switching from anti-IL-5 treatment to dupilumab [93]. We have also reported that patients with severe asthma and high FeNO (>27 ppb) and a low blood eosinophil count (<265 cells/μL) are refractory to treatment with biologics, including anti-IgE Abs or anti-IL-5/IL-5R Abs [25]; these patients are expected to be responsive to dupilumab. Therefore, switching to dupilumab is recommended for patients with severe asthma who are refractory to anti-IL-5 treatment and have FeNO of more than 25 ppb.

## 9. Dupilumab May Suppress Respiratory Infection in Severe Asthma

Dupilumab suppresses investigator-reported respiratory infection events and systemic anti-infective medication use in both patients with asthma and in patients with ECRS [94]. Furthermore, dupilumab is associated with a reduced risk of serious/severe infections and non-herpetic skin infections [95]. Although the mechanisms according to which dupilumab suppresses infection have not been fully clarified, several potential mechanisms have been proposed. First, IL-4/IL-13 increases the viral load of respiratory viruses such as rhinoviruses (RV) in vitro and in vivo [96]. Furthermore, RV infections also trigger IL-33-dependent type 2 inflammation in vivo [97]. Therefore, the inhibition of IL-4/IL-13 may reduce the viral load and thus result in a milder unreported infection. Second, IL-4 contributes to the production of IgE, and dupilumab decreases the concentration of IgE in the serum [67]. IgE is known to increase viral susceptibility in vitro and in vivo [98,99,100,101,102]. For example, the production of the anti-viral cytokine IFN-α from influenza-stimulated plasmacytoid (p)DCs is inversely correlated with the concentration of serum IgE [98]. IgE cross-linking of the peripheral blood mononuclear cells suppresses the RV-induced production of the anti-viral cytokines IFN-α and IFN-λ in asthmatic patients [99]. Furthermore, anti-IgE Ab treatment reduces pDC surface receptor (FcεRIα) expression and restores RV-induced IFN-α production from pDCs [100]. Anti-IgE Abs decrease the duration of RV infection, peak RV shedding, and the frequency of RV illnesses [101]. Moreover, anti-IgE Abs reduce the acute severity of RV-induced asthma exacerbation [102]. Therefore, dupilumab reduces IgE and may increase the IFN production from pDCs and suppress viral-induced asthma exacerbation. Finally, dupilumab suppresses Th2-mediated immune responses and normalizes Th1- and Th17-mediated immune responses, which may contribute to responses to infection.

## 10. The Effects of Dupilumab in Controlling Acquired and Innate Immune Responses and the Prevention of Atopic March

Dupilumab is effective for treating atopic dermatitis [103] and is prescribed in the clinical setting. Proactive therapy for atopic dermatitis suppresses the development of house dust mite-specific IgE [104], suggesting the importance of controlling atopic dermatitis in suppressing allergen transcutaneous sensitization. Therefore, dupilumab is expected to suppress allergen sensitization by suppressing IL-4 signaling, thus suppressing IgE production, and to suppress allergen transcutaneous sensitization by controlling atopic dermatitis. Indeed, dupilumab is found to suppress the serum IgE concentration [67]. Furthermore, dupilumab clinically decreases the numbers of peripheral blood ILC2s in asthma patients when compared to those without dupilumab treatment [105]. Moreover, dupilumab reduces Th2 cell and ILC2 counts and the ILC2/ILC3 ratio in the peripheral blood of patients with atopic dermatitis when compared to their values before dupilumab treatment [106]. A higher ILC2/ILC3 ratio before treatment predicts responsiveness to dupilumab. Therefore, dupilumab can suppress the overall immune response, including acquired immune responses through the suppression of allergen sensitization and Th2 differentiation and innate immune responses through the suppression of ILC2 activation, and thereby suppress the spread of allergen sensitization and/or the progression of atopic march. Indeed, a recent study has reported that dupilumab suppresses the onset of asthma when used to treat atopic dermatitis [107], suggesting that dupilumab can interfere with atopic march in vivo. There is a possibility that the inhibition of IL-4/IL-13 using other approaches, for example, using bacterial lysates or herbal medicines, may suppress transcutaneous sensitization and atopic march, which should be examined in future.

## 11. The Limitations of Anti-IL-4/IL-13 Treatment (Dupilumab)

There are several papers stating that eosinophilic pneumonia and vasculitis-like disease, including eosinophilic granulomatosis with polyangiitis (EGPA), are associated with dupilumab treatment [108,109,110]. Asthma worsening with peripheral blood eosinophilia, eosinophilic pneumonia, or thrombosis may occur after dupilumab administration, even after a good initial response to dupilumab. Potential mechanisms of eosinophilic pneumonia after dupilumab treatment are the inappropriate distribution of the eosinophils due to blocked IL-4/IL-13, drug-induced pneumonia, or the presence of vasculitis-like disease, including EGPA or its pre-stage before dupilumab treatment. Nishiyama et al. reported that serum IL-5 is increased and lung eosinophils are activated in cases of eosinophilic pneumonia after dupilumab treatment, suggesting that IL-5-driven pathology plays a role in its development [110].

Contraindications are those with a history of hypersensitivity to the ingredients of dupilumab. Considering the above background, dupilumab should be used with caution in the active phase of EGPA, although it is not contraindicated. However, if EGPA is stable, its use may be acceptable considering its effect on ECRS and respiratory function.

## 12. Conclusions

IL-4/IL-13 do not directly activate eosinophils; however, they induce eosinophilic airway inflammation via VCAM-1 (for adhesion), CCR3 ligands (for migration), and periostin. Dupilumab decreases asthma exacerbations and increases lung function in moderate to severe asthma. It is particularly effective in patients with elevated peripheral blood eosinophil counts or with elevated FeNO. In addition, dupilumab has been reported to maintain improved lung function and to reduce mucus plugs in severe asthma cases. It is also expected to improve asthma control and exacerbations more in patients with severe asthma with ECRS and to suppress allergen sensitization and the progression of atopic march. Collectively, IL-4/IL-13 appear to play crucial roles in the development of severe eosinophil-predominant asthma.
